# Reversal of a full-length mutant huntingtin neuronal cell phenotype by chemical inhibitors of polyglutamine-mediated aggregation

**DOI:** 10.1186/1471-2202-6-1

**Published:** 2005-01-13

**Authors:** Jin Wang, Silvia Gines, Marcy E MacDonald, James F Gusella

**Affiliations:** 1 Molecular Neurogenetics Unit, Center for Human Genetic Research, Massachusetts General Hospital, Boston MA 02129, USA; 2 Departament de Biologia Cellular i Anatomia Patològica, Facultat de Medicina, Universitat de Barcelona, Barcelona 08036, Spain; 3 Department of Genetics, Harvard Medical School, Boston MA 02115, USA

## Abstract

**Background:**

Huntington's disease (HD) is an inherited neurodegenerative disorder triggered by an expanded polyglutamine tract in huntingtin that is thought to confer a new conformational property on this large protein. The propensity of small amino-terminal fragments with mutant, but not wild-type, glutamine tracts to self-aggregate is consistent with an altered conformation but such fragments occur relatively late in the disease process in human patients and mouse models expressing full-length mutant protein. This suggests that the altered conformational property may act within the full-length mutant huntingtin to initially trigger pathogenesis. Indeed, genotype-phenotype studies in HD have defined genetic criteria for the disease initiating mechanism, and these are all fulfilled by phenotypes associated with expression of full-length mutant huntingtin, but not amino-terminal fragment, in mouse models. As the *in vitro *aggregation of amino-terminal mutant huntingtin fragment offers a ready assay to identify small compounds that interfere with the conformation of the polyglutamine tract, we have identified a number of aggregation inhibitors, and tested whether these are also capable of reversing a phenotype caused by endogenous expression of mutant huntingtin in a striatal cell line from the *Hdh*^*Q111/Q111 *^knock-in mouse.

**Results:**

We screened the NINDS Custom Collection of 1,040 FDA approved drugs and bioactive compounds for their ability to prevent in vitro aggregation of Q58-htn 1–171 amino terminal fragment. Ten compounds were identified that inhibited aggregation with IC_50 _< 15 μM, including gossypol, gambogic acid, juglone, celastrol, sanguinarine and anthralin. Of these, both juglone and celastrol were effective in reversing the abnormal cellular localization of full-length mutant huntingtin observed in mutant *Hdh*^*Q111/Q111 *^striatal cells.

**Conclusions:**

At least some compounds identified as aggregation inhibitors also prevent a neuronal cellular phenotype caused by full-length mutant huntingtin, suggesting that *in vitro *fragment aggregation can act as a proxy for monitoring the disease-producing conformational property in HD. Thus, identification and testing of compounds that alter *in vitro *aggregation is a viable approach for defining potential therapeutic compounds that may act on the deleterious conformational property of full-length mutant huntingtin.

## Background

Huntington's disease (HD) is a severe, dominantly inherited neurodegenerative disorder that typically has its onset in mid-life, though it may occur in the juvenile years or in the elderly, and that produces an inexorable decline to death 10–20 years later [1]. Its cardinal clinical feature is a characteristic motor disturbance involving progressive choreoathetosis, but the disorder also involves psychological changes and cognitive decline. The neuropathological hallmark of HD is the loss of medium spiny striatal projection neurons in a dorso-ventral/medio-lateral gradient that eventually decimates the caudate nucleus, but considerable neuronal loss also occurs in other parts of the basal ganglia and in the cortex [2]. The pathogenic process of HD is initially triggered by an expanded polyglutamine segment near the amino terminus of huntingtin, an ~350 kDa protein whose precise physiological function is uncertain [3]. Huntingtin is required for normal embryonic development and neurogenesis, based on the lethal consequences of mutational inactivation in the mouse [4-6]. By contrast, the HD mutation itself does not impair this developmental activity but rather produces a "gain-of-function" that acts to cause the disorder [7]. Genotype-phenotype studies of HD patients, in comparison with other polyglutamine neurodegenerative disorders, have delineated a number of genetic criteria for the mechanism that triggers HD pathogenesis: 1) a threshold polyglutamine length (within a normal human lifespan); 2) progressive severity with increasing polyglutamine length above the threshold; 3) complete dominance over the wild-type protein; 4) greater dependence on polyglutamine length than on huntingtin concentration (within a physiological range) and 5) striatal selectivity, due to the huntingtin protein context in which the polyglutamine tract is presented [8,9].

The "gain-of-function" due to the HD mutation is thought to lie in a novel conformational property conferred on mutant huntingtin by the expanded polyglutamine tract [10]. This has been supported by *in vitro *studies of a small amino-terminal huntingtin fragment, where an expanded polyglutamine tract promotes self-aggregation in a manner that conforms to the first four genetic criteria [10-12]. The *in vitro *aggregation involves a conformational change of the polyglutamine segment from a random coil to an amyloid structure and is paralleled in cell culture in some ways by the formation of cytoplasmic and nuclear inclusions that also incorporate other proteins [13]. Neuronal inclusions containing amino-terminal fragment have also been detected in HD brain, though their role in pathogenesis remains a matter of debate, as they may occur late in the pathogenic process as a consequence of huntingtin degradation [14].

Precise genetic modeling of HD in the mouse supports the view that *in vivo*, the "gain-of-function" property conferred by the expanded polyglutamine acts within full-length huntingtin to cause abnormalities that do not initially involve formation of an insoluble aggregate [15,16]. Knock-in mice in which the HD mutation has been introduced into *Hdh*, the mouse orthologue, display early biochemical and histological phenotypes that are associated with expression of full-length mutant huntingtin at normal physiological levels and in a normal developmental pattern [7,15-20]. Indeed, the phenotypes associated with expression of full-length mutant huntingtin in these mice, and in neuronal progenitor cells derived from them, also fulfill the genetic criteria for the mechanism triggering HD pathogenesis [15,20-22] One of the earliest phenotypes is the nuclear localization of full-length mutant huntingtin in the nucleus of striatal neurons [16]. Together, the knock-in mouse data suggest that the process of pathogenesis is triggered by the presence of expanded polyglutamine in full-length huntingtin and leads only after many months to the formation of amino-terminal huntingtin fragment and inclusion formation [15].

We have postulated that the same conformational property that promotes aggregation in the context of a small fragment may also act with the context of full-length huntingtin to trigger pathogenesis, possibly by altering huntingtin's interaction with another cellular element. Consequently, we have identified small molecules from the NINDS Custom Collection of bioactive compounds that inhibit *in vitro *aggregation of amino-terminal mutant huntingtin [23]. These have been tested for their ability to reverse the huntingtin localization phenotype associated with full-length mutant huntingtin in cultured striatal progenitor cells from *Hdh *knock-in mice. Our findings indicate that some of these compounds reverse the effects of the expanded polyglutamine in both assays and support the view that some inhibitors of polyglutamine aggregation may lead to viable therapeutics targeted at full-length mutant huntingtin, early in the disease process.

## Results

### Screening for inhibitors of aggregation

**Figure 1:**
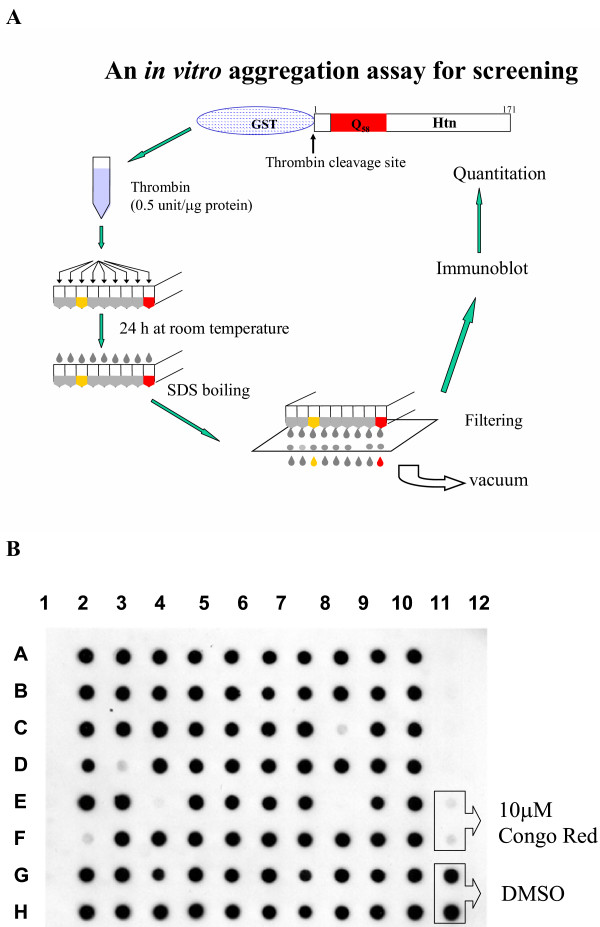
**Schematic diagram of the aggregation screening assay (A) and typical results (B) A**: Scheme of an *in vitro *mutant huntingtin aggregation assay modified for drug screening. In the primary screening, the mixture of fusion protein, GST-Q58-Htn (20 μg/ml) and Thrombin (0.5 unit/μg protein) was immediately distributed into the 96-well plates containing diluted compounds at 40 μl/well. The final concentration of the small compounds is 100 μM. 10 μl of 10% SDS/50 mM 2-mercaptoethanol was added into each well to stop the reaction after 24 hours incubation at room temperature. The aggregates were separated by filtering through a cellulose acetate membrane (0.2 μm). Immunoblotting was done with a specific anti-huntingtin antibody, HP1, followed by incubation with peroxidase conjugated anti-rabbit antibody. The signals of the retained aggregates were scanned and quantified. In the secondary screening, compounds tested positive in were tested at 10 μM and a 45-minute incubation at room temperature was followed after mixing the protein and enzyme. **B**: A typical immunoblot of the huntingtin aggregation inhibitor screening. The aggregates retained on the membrane were visualized by ECL. The intensity of dot reflects the amount of aggregates. The blot shows the positions of each drug in a 96-well plate. Congo Red: positive control (*row *E, *column *12 and *row *F, *column *12). DMSO: negative control (*row *G, *column *12 and *row *H, *column *12). Drugs in wells F2, E4, E9 and D3 are huntingtin aggregation inhibitors.

We have previously demonstrated that, when released from the protection of a GST fusion protein, the amino terminal fragment 1–171 of mutant huntingtin, forms aggregates in a manner consistent with the genetic criteria for the mechanism of HD pathogenesis [10]. We used a modified version of this assay, implemented using a 96-well format ELIFA dot blot apparatus, to screen the NINDS Custom Collection (NCC) which consists of 1040 small bioactive compounds, both FDA-approved drugs and natural products (Figure 1A). The screening was carried out in a blinded fashion as part of the NINDS Neurodegeneration Drug Screening Consortium, with the identities of compounds in the NCC only being made available after completion of the screens [23].

In our primary screen (Figure 1A), GST-Q58-Htn (20 μg/ml) was mixed with thrombin (0.5 unit/μg GST-Q58-Htn) and immediately dispensed into a 96-well PCR plate containing compounds diluted to a final concentration of 100 μM. Incubation was continued for 24 hours at room temperature to allow aggregate formation. The aggregation was stopped by 2% SDS/10 mM 2-mercaptoethanol followed by boiling for 5 minutes. The mixture was filtered through a cellulose acetate membrane by using a 96-well ELIFA dot blot apparatus. The aggregates retained on the membrane were detected and quantified by immunoblotting and subsequent image analysis. A typical immunoblot result is shown in Figure 1B. Congo Red, a known huntingtin aggregation inhibitor, was used as the positive control [24]. 10 μM Congo Red can completely inhibit the Q58-Htn aggregation. DMSO, used for the negative control, had no impact on Q58-Htn aggregation.

**Figure 2:**
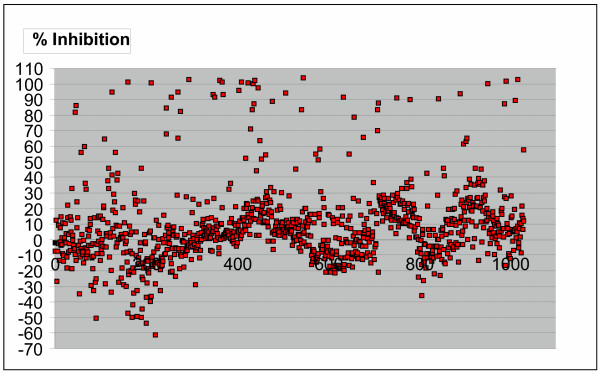
**Distribution of potential huntingtin aggregation inhibitors in the NCC library**. Most compounds show no or little inhibitory effect on huntingtin aggregation. The potential inhibitors are evenly distributed across the whole chemical library. Due to very high background on particular regions of some dot blotting membranes, some compounds (compounds around 200) show a high apparent promotional effect that is artefactual (as shown in ~-70% inhibition). The 8 compounds located in column 5 of plate 9 were missed and were tested in the second screening at 10 μM.

**Figure 3:**
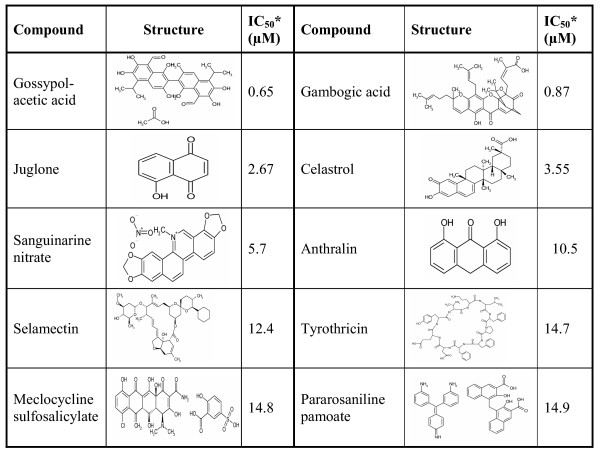
Ten most potent aggregation inhibitors from the NCC library

Potential inhibitors were distributed evenly cross the whole NCC library (Figure 2). Sixty compounds that showed more than 50% inhibitory effect were selected to be retested in a second screen at a lower concentration of 10 μM. The 8 compounds in column 5 of plate 9, were missed in the primary screening at 100 μM, and were therefore also tested in the second screening at 10 μM. In the primary screening, a "hit" could have resulted either from direct inhibition of polyglutamine-induced aggregation or indirectly, by inhibition of the thrombin and consequent failure to cleave GST-Q58-Htn, which does not by itself aggregate. Consequently, the second screening at 10 μM was carried out after thrombin digestion, to eliminate thrombin inhibitors. Western blotting showed that more than 95% of GST-Q58-Htn is cleaved by thrombin (at ratio of 0.5 unit/1 μg protein) within 30 minutes (data not shown). Consequently, the mixture of GST-Q58-Htn and thrombin was preincubated for 45 minutes, followed by centrifugation to remove any aggregates already formed, before adding the test compounds. Nineteen of the compounds tested at 10 μM, showed significant direct inhibitory effects on aggregation. The 10 most potent compounds, corresponding to a 'hit' rate of 1%, are shown in Figure 3.

### Characteristics of aggregation inhibitors

**Figure 4:**
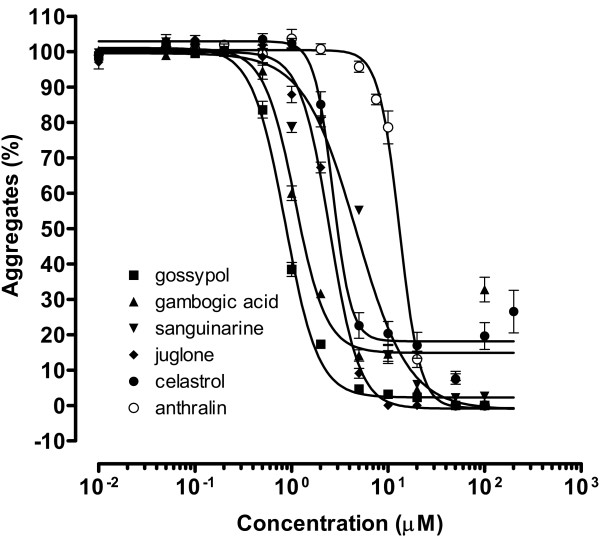
**Dose-response curve of mutant huntingtin aggregation inhibitors**. Compounds were added in 1 μl DMSO to 40 μl 20 μg/ml (0.25 μM) GST-Q58-Htn pre-incubated with thrombin. The reaction was carried out at room temperature for 24 hours. The intensity of each dot of the dot blot was normalized to the DMSO control. Assays were performed twice, in triplicate at each concentration each time (n = 6).

To determine the potency of each inhibitor, we performed dose response assays at concentrations ranging from 0.01 μM to 500 μM. Representative curves for the 6 most potent compounds are shown in Figure 4. Gambogic acid and celastrol showed strong but incomplete inhibition even at the maximum concentration, permitting approximately 20% residual aggregate to form. The average IC_50 _(half-maximal inhibition) values for the most potent 10 compounds, which range from 0.7 to 15 μM, were obtained from at least two independent experiments each (Figure 3). The most effective aggregation inhibitor was gossypol-acetic acid complex, followed closely by gambogic acid, and then juglone, celastrol, and sanguinarine nitrate, which all had IC_50 _values less than 6 μM.

### Effect on striatal cells expressing endogenous full-length mutant huntingtin

**Figure 5:**
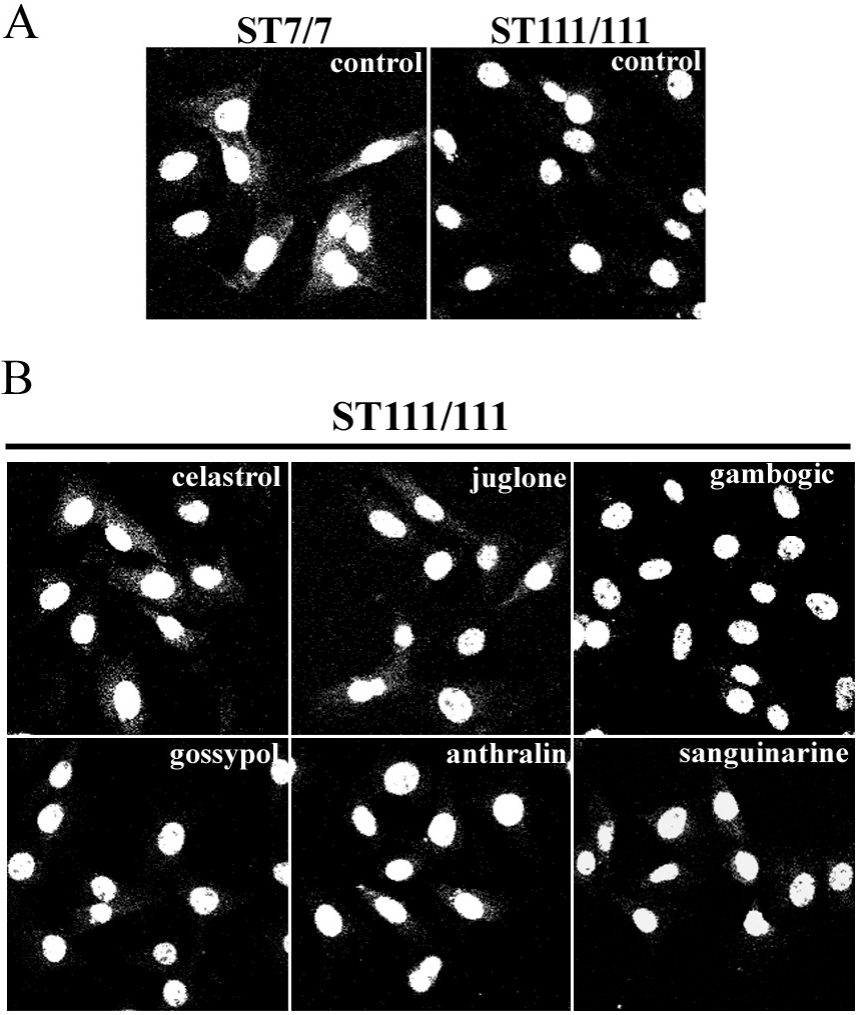
**Localization of huntingtin in wild type and mutant striatal cell from knock-in mice**. Cells were cultured for 48 hours and immunostaining was done with a specific antibody against huntingtin, AP229, followed by Cy3 labeled secondary antibody. The signal was visualized by confocal microscopy (magnification: ×20). A. The localization of full-length huntingtin of striatal cell lines: ST7/7 and ST111/111 with 2.5% DMSO. B. The localization of huntingtin of mutant striatal cell, ST111/111, after treated with drugs at 10 μM.

To test the hypothesis that compounds, which inhibit the aggregation-promoting property of amino-terminal mutant huntingtin will also rescue effects of full-length mutant huntingtin, we tested the top six inhibitors in a striatal cell-based assay. Mutant *Hdh*^*Q111/Q111 *^and wild-type *Hdh*^*Q7/Q7 *^striatal cell lines, ST7/7 and ST111/111, respectively, which have been prepared by transformation with a tsSV40 vector, can be propagated in culture and used for cytological and biochemical comparisons [25]. These cells express full-length mutant or wild-type huntingtin, respectively, with no evidence of truncated amino-terminal fragments, no formation of polyglutamine aggregates and no cell death-producing toxicity. However, like the striatal neurons of *Hdh*^*Q111/Q111 *^knock-in mice, the ST111/111 cells show nuclear staining of huntingtin when tested with an amino-terminal huntingtin antibody that is sensitive to the conformation of the full-length protein (Figure 5A). By contrast, ST7/7 cells expressing wild type huntingtin show both nuclear and cytoplasmic immunostaining with the same huntingtin antibody (Figure 5A). This differential localization phenotype occurs early in the cascade of events detected during the lifespan of *Hdh *knock-in mice, months before the appearance of huntingtin amino-terminal fragment, and fulfills the genetic criteria from genotype-phenotype studies in HD patients, including polyglutamine length progressiveness and striatal specificity, suggesting that it follows from the same property that triggers HD pathogenesis.

This huntingtin localization phenotype was used to monitor the effect of inhibitors in the mutant cells (Figure 5B), and the results are shown in Table 1. About 89% of ST7/7 striatal cells showed both nuclear and cytoplasmic immunostaining signal, while ST111/111 striatal cells showed only nuclear signal (99%). Of the six compounds tested, celastrol and juglone both reversed the mutant phenotype in a dose-dependent manner. Juglone showed no evident cell toxicity up to 10 μM, where 68% of mutant cells had reverted to wild-type phenotype. Celastrol reverted up to 81% of the cells but showed toxicity, killing ~4% of cells at 10 μM. Gossypol acetic acid complex was less effective, but showed no toxicity. At 50 μM, only 15% of the mutant cells displayed the wild-type phenotype. Gambogic acid showed a comparable small effect at 10 μM but was very toxic at high concentration, as all cells were killed at 50 μM. Sanguinarine nitrate and anthralin showed little effect on the huntingtin localization phenotype in this assay.

## Discussion

A dramatic marker of pathology in many neurodegenerative disorders is the appearance of intracellular inclusions in some surviving neurons [13]. In HD, these inclusions stain positively for huntingtin, ubiquitin and a number of other proteins, but are thought to be initiated by the aggregation of an amino-terminal fragment of mutant huntingtin, due to its expanded polyglutamine tract [14,26-28]. A number of model systems have been developed to investigate the polyglutamine-driven aggregation process and its consequences both *in vitro *and *in vivo*, but it remains unclear in HD whether the formation of aggregates plays an essential role in the pathway of pathogenesis or is a downstream by-product of neuronal dysfunction induced by full-length mutant huntingtin [29]. In either event, the search for drugs that alter *in vitro *aggregation of amino-terminal huntingtin fragment is attractive, since the aggregation-promoting physical property exhibits characteristics comparable to the disease-producing property of corresponding human alleles, as defined from genotype-phenotype studies of HD patients. For example, if accumulation of huntingtin inclusions is the proximate cause of neuronal death, compounds that inhibit aggregation would have therapeutic potential. Conversely, if the inclusions are only a downstream marker of the pathogenic process, the same drugs may still have therapeutic potential if they act on the property of full-length mutant huntingtin that triggers pathogenesis. It was with a view to testing whether the *in vitro *aggregation assay could act as a proxy for monitoring the disease-producing property of mutant huntingtin that we undertook this study.

A comparable screening assay to the one used here has been employed to screen a large chemical library and has demonstrated the feasibility of identifying small molecule inhibitors of polyglutamine aggregation, including a family of benzothiazole-related compounds [30]. However, the long, arduous and expensive process of developing compounds for use as drugs in humans prompted us to screen a smaller chemical library biased toward drugs already approved by the U.S. Food and Drug Administration. The aggregation-inhibiting compounds that we identified came from a collection of mostly FDA-approved compounds and bioactive natural products that were specifically assembled for a neurodegenerative disease drug screening consortium supported by the National Institute for Neurological Disorders and Stroke and several disease foundations, including the Huntington's Disease Society of America [23]. The drugs were distributed to 27 different labs for blinded testing in assays of potential relevance to neurodegenerative disease.

Unfortunately, despite the preponderance of FDA approved drugs in the collection, none of our top hits (IC_50 _< 10 μM) is a compound approved for internal use in humans. The most effective inhibitor of aggregation was gossypol-acetic acid complex. Gossypol, a polyphenol found in cottonseed, has been studied extensively as a male contraceptive in China but the World Health Organization has argued against its use because of induced hypokalemia, high toxicity and the risk of permanent sterility [31]. Almost as potent, gambogic acid is a complex ring-structured natural product that is the main active ingredient of gamboge resin from the *Garcinia hanburyi *tree. It has long been used as a pigment for painting and in traditional medicine as a potent purgative. Recently, it was identified in a high-throughput screen as an apoptosis inducer with potential for development as an anti-cancer agent [32]. Juglone is a napthoquinone found in the bark of the black walnut (*Juglans nigra*), which has been used as an herbal medicine for its antihaemorrhagic and antifungal properties [33]. Celastrol is a triterpenoid from the vine *Tripterygium wilfordii *used as an alternative medicine for rheumatoid arthritis that has anti-oxidant, anti-inflammatory activity, immunosuppressive and anti-angiogenic activities. It has been proposed as worthy of exploration as a therapeutic in Alzheimer disease [34]. Sanguinarine, a benzophenanthridine alkaloid from bloodroot (*Sanguinaria canadensis*) has broad antimicrobial and anti-inflammatory, as well as potential anti-angiogenic activity [35]. It has been used as an oral rinse and as a potential antigingivitis/antiplaque agent in toothpaste [36]. Anthralin is a synthetic derivative of chrysarobin, a traditional remedy for various skin ailments from *Andira araroba*, that has been widely used as topical treatment for psoriasis and alopecia areata [37,38].

Although none of these bioactive compounds is a candidate for immediate human trials, they provided a means to test whether compounds that inhibit polyglutamine aggregation might also block the neuronal phenotype caused by an elongated polyglutamine tract in full-length huntingtin. While we do not have a direct physical measure of huntingtin conformation and it remains possible that compounds could reverse the cellular phenotype by different pathways, our finding that juglone and celastrol, two drugs of different structure selected as hits in our primary aggregation assay, are both effective at restoring cytoplasmic huntingtin staining in the striatal cell assay suggests that they both act via a conformational property of mutant huntingtin. That gossypol, gambogic acid, sanguinarine and anthralin were not effective could be due to any of a number of reasons, including cellular uptake, toxicity, interaction with other cellular components, etc. However, it may also indicate that these compounds do not directly modify the conformational property of mutant huntingtin, but instead block a different step in the *in vitro *aggregation process or that they do so by a different physical effect than juglone and celastrol. A detailed analysis of structure-activity relationships using structurally-related compounds and testing *in vivo *in knock-in mice for their ability to reverse the cascade of mutant huntingtin-associated phenotypes will be needed to adequately assess the potential of any of these different types of compounds for testing in HD clinical trials.

The remaining compounds identified as weaker aggregation blockers in our primary screen include selamectin, a veterinary anti-parasitic [39], pararosaniline pamoate, a treatment for schistosomiasis [40], tyrothricin, a cyclic peptide antibiotic, and meclocycline, a tetracycline-related antibiotic. The latter is of particular interest since it was the most potent of several tetracycline-related antibiotics present among the NCC compounds, including tetracycline, chlortetracycline, demeclocycline, doxycycline, methacycline, oxytetracycline and notably, minocycline, which has been proposed as a therapeutic in HD and other neurodegenerative disorders. Minocycline is an FDA approved antibiotic used for a variety of infections that has variably been reported to improve symptoms in the R6/2 exon 1 overexpression HD model [41-44]. It has anti-inflammatory and anti-apoptotic activity that has been proposed to involve several potential mechanisms of action. In our hands, minocycline is a weak inhibitor of polyglutamine aggregation with an IC_50 _of 43 μM (unpublished data). This is consistent with the inhibitory effect on huntingtin exon 1 aggregation reported previously at 30 μM in long-term hippocampal slice cultures from the R6/2 mouse [44]. Two safety and tolerability studies of minocycline in human HD are completed [45,46] and can be expected to lead to efficacy trials earlier than any trials for strong aggregation inhibitors. However, it is conceivable that long-term, low level inhibition of mutant huntingtin's aggregation-promoting conformational property, independent of minocycline's anti-apoptotic activity, may be sufficient to alter detectably the timing of disease onset or early progression. If the hoped for positive results are obtained in minocycline HD trials, this alternative mechanism should be considered since it would have implications for testing of meclocycline and for assessing the potential trade-off between potency and toxicity in choosing other aggregation inhibitors as potential long-term therapeutics.

Interestingly, the same set of 1040 NCC compounds were screened for their ability to block toxicity in a PC12 cellular assay where induced expression of huntingtin exon 1 encoding 103 glutamines leads to the accumulation of aggregates and rapid cell death [47]. Although eighteen compounds were found to be completely protective, none was among our hits, suggesting that the mechanism of polyglutamine toxicity in the PC12 cells is fundamentally different than the mechanism(s) involved in the *in vitro *aggregation assay. Among a secondary class of partially protective compounds in the PC12 assay, only celastrol overlapped with our hits. The NCC compounds were also screened in a cellular assay in HEK 293T cells expressing androgen receptor with 112 glutamines [48]. In this model for spinal bulbar and muscular atrophy, accumulation of intracellular inclusions, accompanied by caspase 3 activation, is followed by cell death within 72 hours. Twenty compounds that blocked caspase 3 activation included celastrol, gambogic acid, sanguinarine and tyrothricin, though all but sanguinarine showed toxicity. The major finding from this assay was that several cardiac glycosides were protective, presumably by a different mechanism than our hits. Indeed, although most of the assays in the NINDS consortium involved disorders associated with protein aggregation, including various polyglutamine disorders, amyotrophic lateral sclerosis and Parkinson disease, there was a remarkable lack of overlap in hits suggesting that the individual assays targeted fundamentally different mechanisms. A possible exception was celastrol, which was found as a hit in our aggregation assay, the two assays noted above, and other assays which will be discussed in a summary article describing the consortium.

## Conclusions

The identification and further characterization of chemical inhibitors of *in vitro *aggregation of an amino-terminal fragment of mutant huntingtin offer promise for the development of potentially therapeutic compounds that also target the deleterious conformational property of full-length mutant huntingtin.

## Methods

### Chemical library, enzymes and antibodies

A library containing 1040 small chemical compounds consisting of FDA-approved drugs and bioactive natural products, the National Institute of Neurodegenerative Diseases and Stroke Custom Collection (NCC), was provided in thirteen 96-well plates by MicroSource Discovery Systems, Inc (Gaylordsville CT). The complete list of NCC compounds is available [49]. All compounds were dissolved in 100% DMSO at a concentration of 10 mM. Thrombin was purchased from Amersham Pharmacia Biotech (Piscataway NJ). Anti-huntingtin antibody HP1, used in the screening assay was described by Persichetti et al. [50]. AP229 used in the cell-based assay was previously described and was a gift of Dr. A.H. Sharp [25].

### GST-huntingtin construct and expression

A recombinant pGEX-2TK expression vector with cDNA fragment encoding amino terminal 171 amino acids of human huntingtin with a polyglutamine tract of 58 was used to prepare the GST-Q58-htn fusion protein. The GST-Q58-htn was overexpressed in BL21 cells and purified by affinity chromatography over glutathione-sepharose 4B beads (Amersham Pharmacia Biotech). The purified proteins were stored at concentration of 2.0 mg/ml at -80°C.

### Aggregation assay

For primary screening of the chemical library, 1 μl of 4.0 mM small compound stock, diluted from the original plates, was placed in wells of 96-well plates. The fusion protein, GST-Q58-Htn was mixed with thrombin (0.5 unit/1 μg protein) at a concentration of 20 μg/ml in a buffer of 50 mM Tris-HCl, pH 8.0, 100 mM NaCl, 2.5 mM CaCl_2_, 1.0 mM EDTA. The mixture was immediately distributed into the 96-well plates containing diluted compounds at 40 μl/well and mixed well. The final concentration of the small compounds was 100 μM. After 24 hours incubation at room temperature, the reaction was stopped by adding 10 μl of 10% SDS/50 mM 2-mercaptoethanol to each well followed by boiling in a PCR machine for 5 minutes. The mixture from each well was filtered through a cellulose acetate membrane ((0.2 μm, GE Osmonics labstore, Minnetonka MN) by using a 96-well ELIFA (Pierce Biotech). The aggregates retained on the membrane were detected by a specific anti-huntingtin antibody, HP1 (diluted 1:1000), followed by incubation with peroxidase conjugated anti-rabbit antibody (diluted 1:10,000, Sigma). Signals from SDS insoluble aggregates were scanned and quantified by using ImageMaster Totalab image analysis software (Amersham Pharmacia Biotech). In the secondary screening, all steps were the same except the final concentration of compound in each well was reduced to 10 μM and the GST-Q58-Htn/thrombin mixture was preincubated for 45-minutes at room temperature and clarified by centrifugation at 28,000 × g for 30 minutes before being added to the test wells. For IC_50 _determinations, the *in vitro *aggregation assay and signal quantification were performed as in the second screening but varying the final concentration of input drug. The data for each inhibitor were obtained from at least two independent experiments in which every sample was analyzed in triplicate with Prism 3.0 software (Graphpad Software, Inc., San Diego, CA).

### Striatal cell line assay

Conditionally immortalized wild-type *Hdh*^*Q7/Q7 *^striatal neuronal progenitor cells (ST7/7) expressing endogenous normal huntingtin, and homozygous *Hdh*^*Q111/Q111 *^striatal neuronal progenitor cells (ST111/111), expressing endogenous mutant huntingtin with 111-glutamines, have been described previously [25]. The striatal cell lines were grown at 33°C in Dulbecco's modified Eagle's medium (DMEM) supplemented with 10 % fetal bovine serum (FBS), 1% nonessential aminoacids, 2 mM L-glutamine and 400 μg/ml G418 (Geneticin (GIBCO-BRL, Life Technologies, Gaithersburg, MD).

In the immunofluorescence and confocal analysis experiment, wild-type ST7/7 and homozygous mutant ST111/111 cells, grown to confluence on glass coverslips, were treated with 6 different drugs at four different concentrations (0.5, 5, 10 or 50 μM) for 30 min at 33°C. After the treatment the drugs were removed and the cells washed twice in PBS. The cells were then fixed by incubation in 4% formaldehyde for 15 min, permeabilized by 0.1% Triton X-100 for 5 min and incubated for 30 min with blocking solution (1% bovine albumin in PBS). Coverslips were then incubated in primary antibody (AP229 1:500 dilution) for 2 h at room temperature and washed three times in PBS before a further 1 h in blocking solution containing the secondary antibody (goat anti-rabbit Cy3, Jackson ImmunoResearch, West Grove, PA. USA). After three washes in PBS coverslips were mounted onto glass slides with Vectashield (Burlingame, CA. USA) and the images were analyzed with a laser confocal microscope (Bio-Rad, Hercules, CA. USA) using 20 × objective. Cell death was quantified by scoring the percentage of cells with apoptotic nuclear morphology, i.e. condensed or fragmented nuclei, under the confocal microscope. In each case five to ten randomly selected fields were counted, comprising at least 200 cells, and each experiment was repeated 3 times.

## Authors' contributions

JW carried out the library screen and aggregation assays. SG carried out the cell-based assays. MEM and JFG contributed to the conception of these studies. JW and JFG drafted the manuscript and MEM and SG contributed to its final version. All authors read and approved the final manuscript.

## Figures and Tables

**Table 1:** Percentage of ST111/111 cells showing cytoplasmic/nuclear huntingtin localization comparable to wild-type ST7/7 cells in response to chemical treatment*

**Compounds**	**Concentration (μM)**			
	
	0.5	5	10	50
Gossypol-acetic acid	0	4.5 ± 4.3%	3.5 ± 3.5%	15.9 ± 11.1%
Gambogic acid	1 ± 2.6%	0	2.1 ± 4.6%	Toxic (100%)
Juglone	10.4 ± 12.4%	37.9 ± 19.9%	68.8 ± 18.4%	N.A.
Celastrol	8.7 ± 8.7%	69.2 ± 3.8% (1%)	78.1 ± 3.5% (4%)	N.A.
Sanguinarine	0 (4.3%)	3.7 ± 5.2% (5.5%)	0 (22.5%)	N.A.
Anthralin	4.6 ± 7.6%	6.6 ± 9.7%	9.6 ± 12.1%	11.8 ± 14.4%

ST111/111 cells with both nuclear and cytoplasmic staining were counted, as well as total cells and dead cells. The effectiveness of the drugs was measured by percentage of cells with nuclear and cytoplasmic staining versus total cells. In untreated cultures, 89% of ST7/7 striatal cells show both nuclear and cytoplasmic immunostaining, while only 1% of ST111/111 striatal cells show this pattern. Entries in the table represent the average values from 5–10 different microcope fields ± standard deviation. Numbers in brackets indicate percentage of dead cells in cases where toxicity was observed.
